# Supplementary figures and images for: An Unusual Case of Collision Testicular Tumor in a Female DSD Dog
Source: Vet Sci. 2023 Mar 27;10(4):251. doi: 10.3390/vetsci10040251 (PMC10144422; doi:10.3390/vetsci10040251)

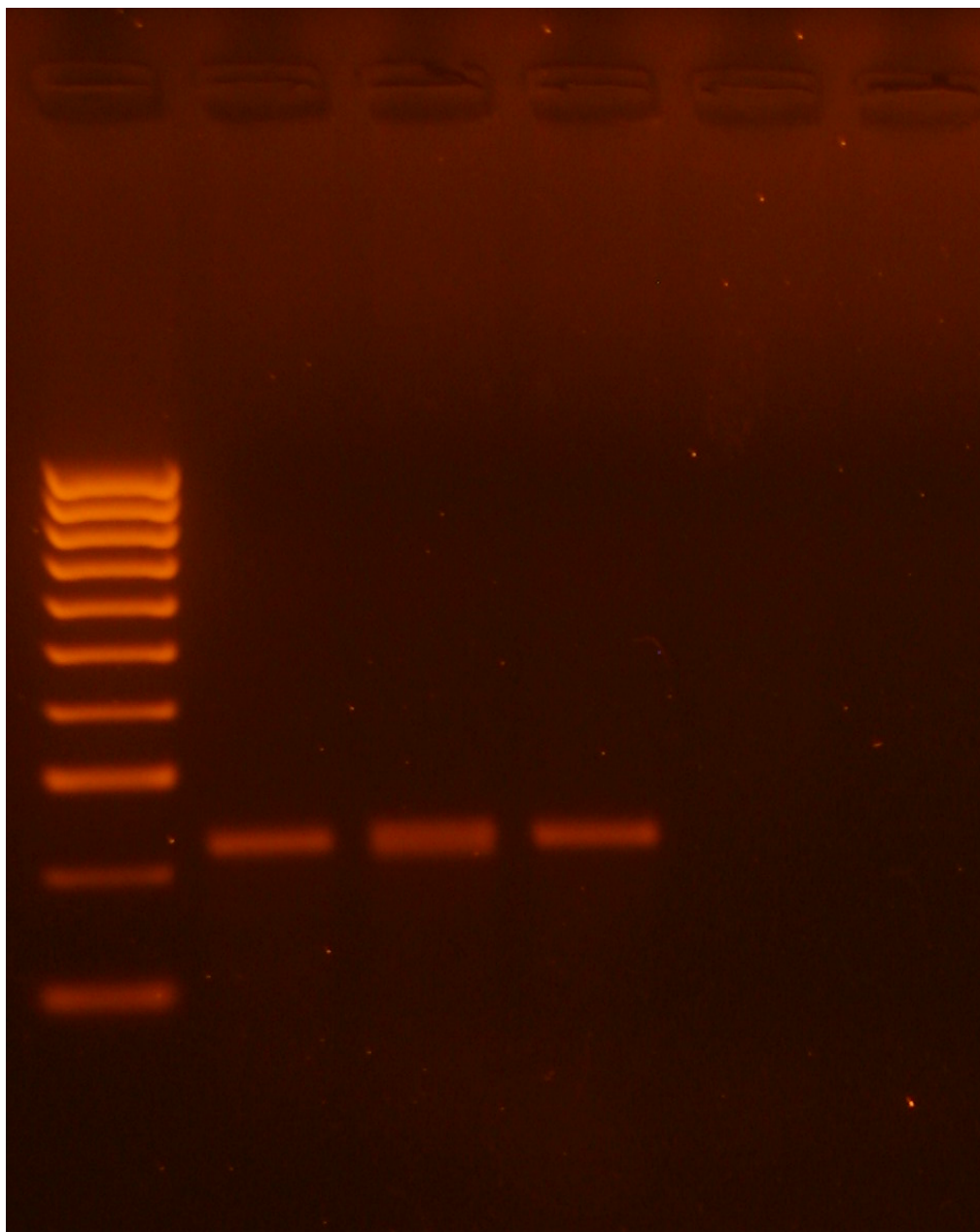

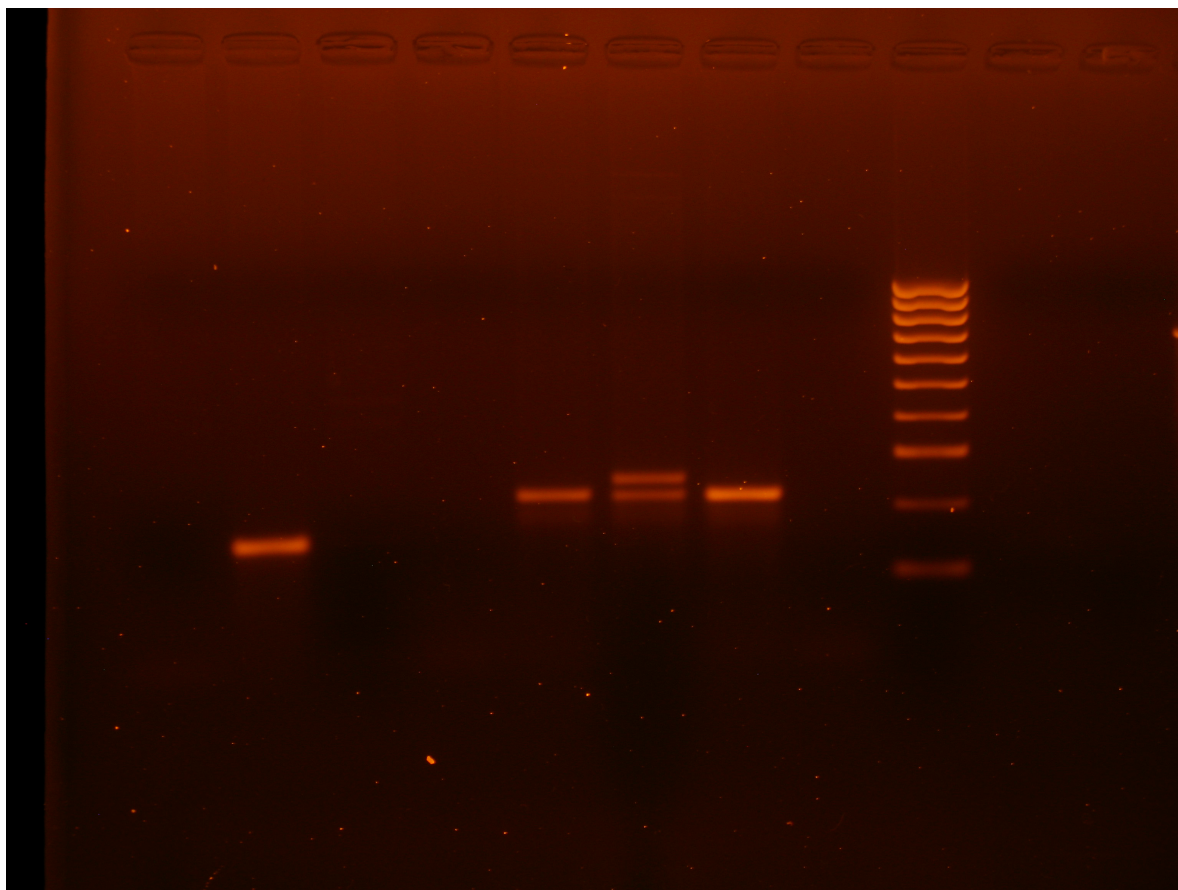

**Figure S1.** Original images for Figure 3.

Supplement: Supplementary file 1 [file vetsci-10-00251-s001.zip › vetsci-2162073-Figure S1.pdf]
